# A rolling phenotype in Crohn's disease

**DOI:** 10.1371/journal.pone.0174954

**Published:** 2017-04-06

**Authors:** James Irwin, Emma Ferguson, Lisa A. Simms, Katherine Hanigan, Franck Carbonnel, Graham Radford-Smith

**Affiliations:** 1 Inflammatory Bowel Diseases Research Group, QIMR Berghofer Medical Research Institute, Brisbane, Australia; 2 Department of Gastroenterology and Hepatology, Royal Brisbane and Women's Hospital, Brisbane, Australia; 3 School of Medicine, The University of Queensland, Brisbane, Australia; 4 Service de Gastroentérologie, Hôpitaux Universitaires Paris Sud, site de Bicêtre Assistance Publique-Hôpitaux de Paris, France; 5 CESP, Fac. de médecine—Univ. Paris-Sud, Fac. de médecine—UVSQ, INSERM, Université Paris-Saclay, Villejuif, France; University Hospital Llandough, UNITED KINGDOM

## Abstract

**Background and aim:**

The Montreal classification of disease behaviour in Crohn's disease describes progression of disease towards a stricturing and penetrating phenotype. In the present paper, we propose an alternative representation of the long-term course of Crohn’s disease complications, the rolling phenotype. As is commonly observed in clinical practice, this definition allows progression to a more severe phenotype (stricturing, penetrating) but also, regression to a less severe behaviour (inflammatory, or remission) over time.

**Methods:**

All patients diagnosed with Crohn's Disease between 01/01/1994 and 01/03/2008, managed at a single centre and observed for a minimum of 5 years, had development and resolution of all complications recorded. A rolling phenotype was defined at each time point based on all observed complications in the three years prior to the time point. Phenotype was defined as B1, B2, B3, or B23 (penetrating and stenotic). The progression over time of the rolling phenotype was compared to that of the cumulative Montreal phenotype.

**Results:**

305 patients were observed a median of 10.0 (Intraquartile range 7.3–13.7) years. Longitudinal progression of rolling phenotype demonstrated a consistent proportion of patients with B1 (70%), B2 (20%), B3 (5%) and B23 (5%) phenotypes. These proportions were observed regardless of initial phenotype. In contrast, the cumulative Montreal phenotype progressed towards a more severe phenotype with time (B1 (39%), B2 (26%), B3(35%) at 10 years).

**Conclusion:**

A rolling phenotype provides an alternative view of the longitudinal burden of intra-abdominal complications in Crohn's disease. From this viewpoint, 70% of patients have durable freedom from complication over time (>3 years).

## Introduction

Crohn's disease is a chronic inflammatory condition affecting the human gastrointestinal tract. Its natural history over time has been described as episodes of disease activity separated by periods of wellness.[1] It is characterized by chronic inflammation, mucosal and sub-mucosal ulceration and fibrosis. These processes cause symptoms of abdominal pain, diarrhoea, bleeding and anaemia, malnutrition, and can cause bowel stenosis, bowel obstruction, bowel perforation and internal fistula formation. Such complications are major events in a patient's disease course.[2,3] Descriptions of how often and when these complications occur for a cohort of patients with Crohn's disease are used to provide patients and clinicians insight into what to expect from the disease.

In 1998 a working group described a classification system for patients with Crohn's disease, with the goal of standardizing disease description in research cohorts, and allowing translation of research findings to clinical practice.[4] The Vienna classification described disease location (L1 ileal, L2 colonic, L3 ileocolonic, L4 upper gastrointestinal) and disease behaviour (B1 inflammatory, B2 stricturing, and B3 penetrating–both internal and perianal), and age at onset of disease (A1 ≤ 40 years, A2 >40). In 2002 a seminal paper on the longitudinal progression of disease behaviour in patients with Crohn's disease was published by Cosnes et al, describing a cumulative Vienna classification.[5] This analysis was performed considering classification to be hierarchical (B1 < B2 < B3), and irreversible. This interpretation of disease behaviour meant that a patient with a bowel stenosis and a perianal fistula, complications which qualify as B2 and B3 disease behaviour respectively, was considered to have a B3 phenotype. Additionally, after resolution of a complication (eg. perianal fistula) which qualified a patient as having a B3 phenotype, classification remained B3 indefinitely. These two aspects of how longitudinal phenotype was defined dictated that observed disease behaviour for individual patients either remained static, or progressed from B1 to B2, B1 to B3, or B2 to B3 with time.

A further consensus disease classification was made in Montreal in 2002.[6] At this time perianal disease was considered to hold different prognostic meaning to internal penetrating disease (perforation/abscess/fistula formation), and was recorded separately.[7] Disease behaviour by the Montreal classification system was defined as either B1 (inflammatory), B2 (stricturing) or B3 (internal penetrating). Analyses of longitudinal disease progression using the cumulative Montreal classification have demonstrated a similar progression towards a more severe phenotype.[8, 9] However, due to the exclusion of perianal disease in the definition of B3 behaviour, the proportion of patients that progress to a B3 classification is less than when the Vienna classification system was used, showing that the representation of the disease is highly dependent of the initial assumption.

These data are used by clinicians to assess risk of future stenosis or internal penetrating complications for individual patients, and to inform patients of what they may expect over the course of their disease. We were struck by the discord between the cumulative Montreal phenotype, and the longitudinal prevalence of internal penetrating or stenotic complications (IPSCs) in our cohort of patients with Crohn's disease. We consider that this is due to the cumulative Montreal phenotype being described as an irreversible hierarchy, which may bias the natural history of Crohn's disease as more severe than many patients experience. This is particularly so for patients who suffer a disease complication at presentation, and then no further subsequent complication.

With two small changes to the definition of disease behaviour, longitudinal phenotype may be observed in a way which is more representative of the burden of IPSCs over time. This article describes a definition of longitudinal phenotype in CD which provides insight into the longitudinal burden of IPSCs.

## Materials and methods

This was a longitudinal observational single centre cohort study. All patients with a diagnosis of Crohn's disease made between 1st January 1994 and 31st March 2008, and managed longitudinally at the Inflammatory Bowel Diseases Unit at the Royal Brisbane and Women's Hospital (RBWH, Brisbane, Australia), were invited to participate in a research programme. Clinical data were recorded at baseline, and further clinical data were recorded longitudinally.

### Inclusion and exclusion criteria

Patients with less than 5 years of clinical follow-up were not included in this study.

### Definitions

#### Diagnosis of CD

The diagnosis of Crohn's disease was made by the treating physician. Patients were required to meet the Lennard-Jones criteria.[10] However, in the absence of a bowel resection specimen we consider the Lennard-Jones criteria are often difficult to meet, due to the inability to demonstrate sub-mucosal fissuring ulceration, or sub-mucosal fibrosis, on mucosal biopsy. Patients without a surgical specimen at diagnosis were required to show evidence of chronic inflammation on mucosal biopsy, with a distribution of affected bowel consistent with Crohn's disease, in the setting of abdominal symptoms attributable to inflammation of duration greater than 3 months.

#### Description of complications

IPSCs and how they were observed are described in Table 1. Complications that were consequent to a surgical procedure were not considered to be IPSCs.

**Table 1:** Identification of Internal Penetrating or Stricturing Complications.

Complication	Modality	
Stenosis	Endoscopy	Narrowing of bowel lumen unable to be passed by endoscope
	Surgery	Macroscopic stenosis of bowel lumen identified at surgery or on pathological specimen
	Radiology	Luminal narrowing of bowel with prestenotic dilation to greater than 2.5cm
Perforation	Surgery	Phlegmon or extraluminal collection
	Radiology	Phlegmon, extraluminal collection, free air under the diaphragm
Fistula	Endoscopy	Internal opening of fistula visible
	Surgery	Macroscopic fistula between two hollow viscera or the skin, identified at surgery or on pathological specimen
	Radiology	Fistula tract evident between two hollow viscera or the skin
	Clinical examination	Cutaneous fistula tract evident on skin (not perianal)

Objective clinical data were recorded retrospectively in a codified longitudinal database by two clinicians (JRI, EF). Objective clinical data included all macroscopic and histological findings at ileocolonoscopy, colonoscopy, flexible sigmoidoscopy, oesophagogastroduodenoscopy, all macroscopic surgical findings at laparotomy and laparoscopy, all histology of surgical specimens, and all findings on computed tomogram (CT) and magnetic resonance imaging (MRI). In the case of enterocutaneous fistulae, clinical examination findings were also recorded. The development of a complication was considered to occur at its first observation with any modality. In this study, the resolution of complication was defined clinically. A complication was considered resolved after the passing of two years of clinical follow-up, without further clinical event related to the complication. This definition of resolution of complication was used regardless of whether an interventional procedure (surgical, endoscopic, interventional radiology) was performed to effect resolution. We consider that a timeperiod of two years was long enough for a severe, unresected CD lesion (stricture, fistula), to manifest.

#### Description of disease behaviour classification

At each time point, for each patient, disease behaviour was defined based on the presence or absence of either stricturing or internal penetrating disease over the preceding 3 years. Disease behaviour was stratified into four levels: inflammatory (B1), stricturing (B2), internal penetrating (B3) and stricturing and internal penetrating (B23). This definition is not hierarchical, and simply describes the presence or absence of IPSCs.

#### Medication use

A medication was considered to be taken after 6 months of use.

### Ethical considerations

Ethical approval for the study was obtained through the Royal Brisbane and Women's Hospital ethics committee. All participating patients provided written consent to take part in the research programme.

### Statistics

This analysis was performed using the R statistical computing environment.[11] Count data are presented as a median and an intraquartile range.

## Results

369 patients were reviewed. 20 left the area during the study period, and 44 were lost to clinical follow-up. 305 patients were included in the analysis. Baseline demographics are shown in Table 2. 144/305 patients were treated with azathioprine for a minimum of 6 months, 127/305 with 6-mercaptopurine, 56/305 with methotrexate, 88/305 with infliximab and 64/305 with adalimumab. 34 patients were treated with no immunomodulator or biologic therapy over their disease course.

**Table 2:** Demographic data.

Age at diagnosis (median and IQR, years)	24.2 (18.7–33.9)
Female	55.1%
Caucasian Australian	86.9%
Smoker	50.2%
Family history of IBD	27.2%
Follow-up (median and IQR, years)	10.02 (7.03–13.80)
Montreal Location at Diagnosis	
L1	130
L2	60
L3	111
No macroscopic ileocolonic disease	4
Perianal disease at diagnosis	29

IBD = inflammatory bowel disease

IQR = intraquartile range

L1 = ileal, L2 = colonic, and L3 = ileocolonic location of disease.

A median of 3 (IQR 2–5) ileocolonoscopies, 1 (IQR 0–2) CT scans, 1 (IQR 0–2) MRI scans, and 1 (IQR 0–1) intra-abdominal surgical procedures were performed per patient over a median observation time of 10.02 years (IQR 7.29–13.71). 130 patients suffered no complication over their observation period, 109 had one complication, 54 had two complications, 22 had three, 8 had four and and 1 patient had five complications. These were made up of 265 episodes of bowel stenosis, 106 bowel perforations, and the formation of 68 internal fistulae were observed. 82 of these were combination events (stenosis and fistula/perforation observed). Resolution was observed in 261 of 320 events. Median time from first observation to resolution of each complication was 2 years (IQR 2–2.54) for stenoses, 2 years (IQR 2–2.58) for fistula/perforation events and 2.30 years (IQR 2.01–3.06) for combination events. Resolution followed surgery in 136 complications, and occurred without surgical intervention in 125 complications.

For the 125 complications which resolved without surgery, 81 patients took a thiopurine, 11 methotrexate, and 23 a biologic (either infliximab or adalimumab) between occurrence of the IPSC and its resolution. 32/125 patients took no medications over this period. For those that took medication following their conservatively managed IPSC, duration of medication use given as a proportion of the resolution period were (lower quartile/median/upper quartile) 0.50/0.83/1.00 (thiopurine), 0.32/0.71/1.00 (methotrexate) and 0.25/0.53/0.89 (biologic medications).

**Fig 1:**
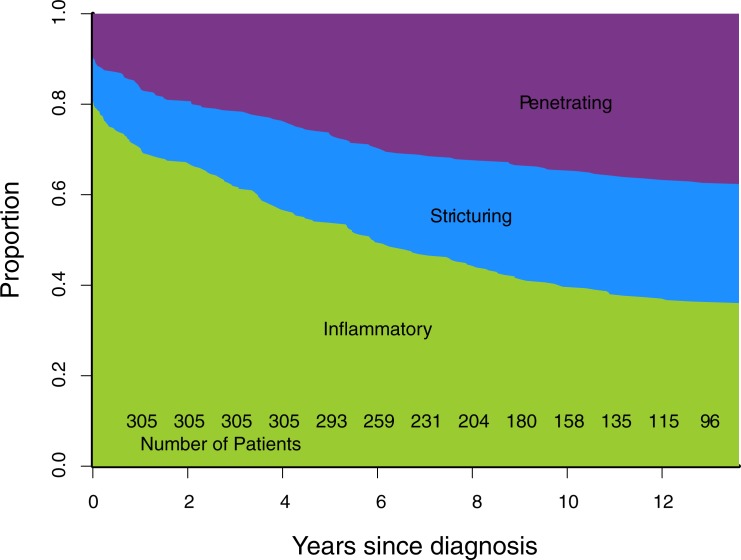
Cumulative Montreal phenotype (B1 inflammatory, B2 stricturing, B3 penetrating).

**Fig 2:**
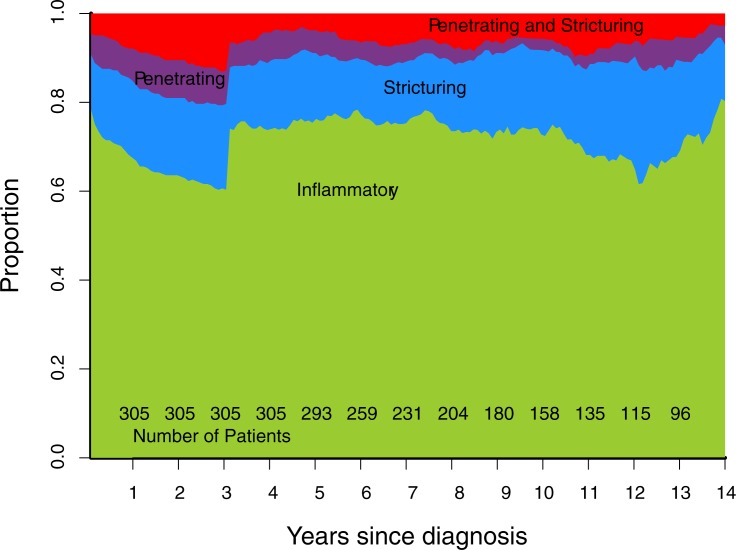
Rolling phenotype (B1 inflammatory, B2 stricturing, B3 penetrating, B23 stricturing and penetrating).

**Fig 3:**
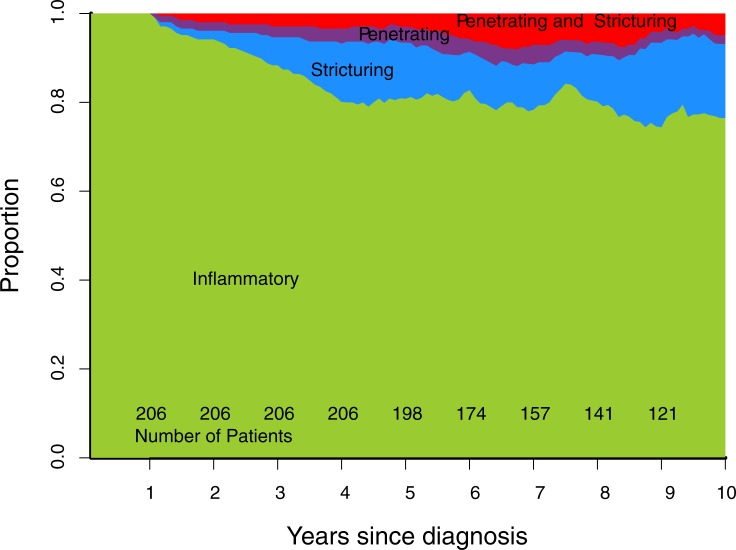
Rolling phenotype, only patients with B1 phenotype within one year of diagnosis. (B1 inflammatory, B2 stricturing, B3 penetrating, B23 stricturing and penetrating).

**Fig 4:**
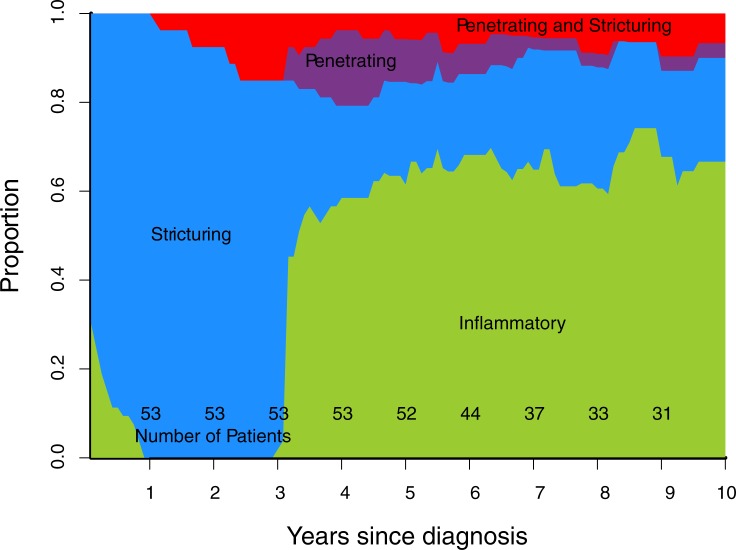
Rolling phenotype, only patients with B2 phenotype within one year of diagnosis. (B1 inflammatory, B2 stricturing, B3 penetrating, B23 stricturing and penetrating).

**Fig 5:**
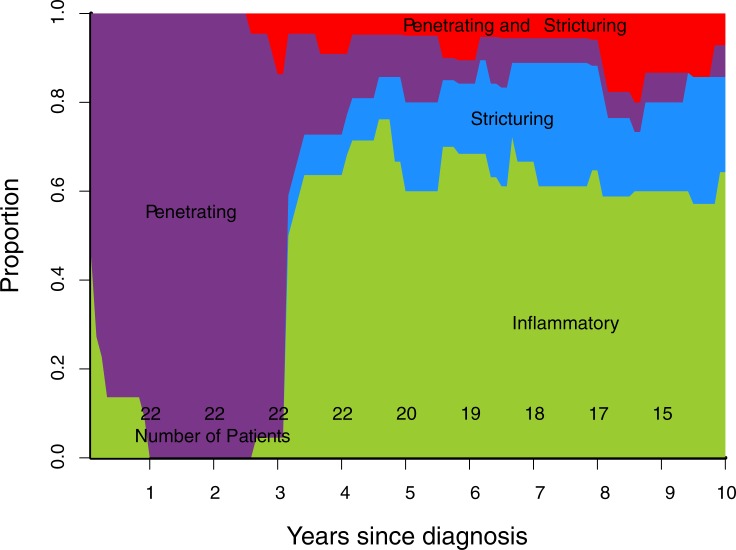
Rolling phenotype, only patients with B3 phenotype within one year of diagnosis. (B1 inflammatory, B2 stricturing, B3 penetrating, B23 stricturing and penetrating).

**Fig 6:**
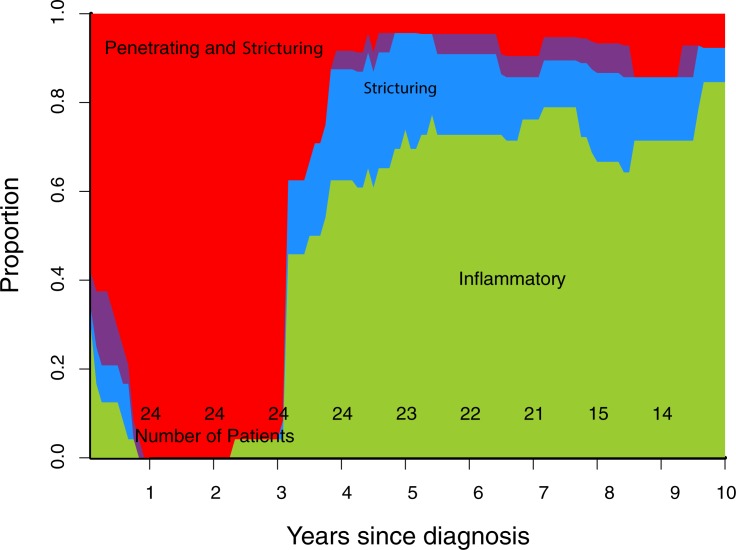
Rolling phenotype, only patients with B23 phenotype within one year of diagnosis. (B1 inflammatory, B2 stricturing, B3 penetrating, B23 stricturing and penetrating).

Cumulative progression of Montreal disease behaviour (Fig 1), progression of rolling phenotype (Fig 2), and progression of rolling phenotype stratified by phenotype within one year of diagnosis (Figs 3–6) are shown below.

## Discussion

The rolling phenotype provides an alternative viewpoint of longitudinal phenotype change in patients with CD.We believe this provides patients and clinicians with a useful insight into long term outcome.

The proportion of patients in our cohort who progress to B2 or B3 phenotype as described by the cumulative Montreal phenotype is similar to published cohorts.[8, 9] The proportion of patients with L1/L2/L3 Montreal disease location at diagnosis (0.43/0.20/0.35) is similar to published cohorts.[2, 5, 9, 12] We consider that our cohort of patients with CD is similar in make-up to these published cohorts, and that the observations we have made are likely to translate to such cohorts.

The progression towards penetrating phenotype seen in the cumulative Montreal phenotype is not observed using a rolling phenotype. Instead, the proportion of patients observed with each classification (B1, B2, B3, B23) remains constant over time. This occurs because the number of patients developing new complications is matched over time by an equal number of patients in whom complications resolve. Put another way, a rolling phenotype does not describe the proportion of patients in a cohort who have ever suffered a stricturing or internal penetrating complication. Instead, it describes a measure of the prevalence of these complications over time.

A rolling phenotype shows a sharp change towards a less severe classification at 3 years (Fig 2). This is inherent in its definition at each time point: 'the presence of any IPSC during the previous 3 years'. In our cohort, as observed in most other cohorts, there is a high frequency of complications observed at diagnosis. All those who suffer a complication at diagnosis, and then none subsequently, revert to B1 or inflammatory phenotype, 3 years after the presenting complication resolves. A rolling phenotype illustrates this observation graphically.

In our cohort there was a predominance of stricturing complications over time, an observation which is described by a rolling phenotype. This observation is not made by the cumulative Montreal phenotype, which describes a progression to internal penetrating phenotype in our cohort. Two aspects of the definition of the cumulative Montreal phenotype contribute to this difference. Firstly, because the Montreal phenotype is hierarchical, patients who suffer both a stricture and a fistula/perforation at the same time are classified as B3 (internal penetrating). Secondly, because the cumulative Montreal phenotype is irreversible and hierarchical, a patient who suffers an internal penetrating complication early in their disease course is always classified with B3 behaviour, even if he/she suffers a subsequent stenotic complication.

Stratifying the cohort by Montreal classification within one year of diagnosis demonstrates that over time B1 and B2 phenotypes are the most common regardless of phenotype in first year (Figs 3–6). This contrasts with published data suggesting phenotype predicts future complications, particularly that penetrating phenotype predicts further penetrating complications.[13] A potential explanation for this discrepancy is that 30% of stenosing events in our cohort were observed on MRI, CT or ileocolonoscopy, but did not lead to surgery. If a similar proportion of stenosing events occurred in the Greenstein cohort they would not have been captured by the recorded outcome of surgery.

These complications have been observed in a cohort of patients who have been treated with immunomodulators and biologic medications. Historical cohorts have had less immunosuppression prescribed.[14,15] Data showing remission induction and maintenance of remission–as defined by the Crohn's Disease Activity Index (CDAI)–are robust for these medications.[16–23] However, there are no clear data demonstrating that immunomodulation decreases the risk of stenosing or internal penetrating complications.[14] Our current treatment paradigm is that immunomodulatory medical therapy reduces inflammation, which reduces tissue damage and associated IPSCs. These data are taken from Crohn's disease patients who are offered medical therapy, and apply to such patients.

Our cohort, managed in a centre which provides both secondary and tertiary inflammatory bowel disease care, is likely to include a higher proportion of patients with IPSCs. It is likely that the prevalence over time of non-inflammatory phenotypes (B2, B3, B23) in a population based cohort would be lower. When translating these data to patients managed at non-tertiary referral centres, one may lean towards increasing the estimate of a complication-free disease course over time, to a figure higher than the 70% we observed.

The cumulative Montreal phenotype gives a negative impression that the majority of patients with CD progress to a complication with time. This is a true representation of the data, however is not representative of the burden of IPSC over the course of a patient's disease. Indeed, it observes no difference between patients in whom an IPSC resolves and those in whom they do not. In contrast, the rolling phenotype provides a more positive, and somewhat realistic, outlook: that over time many IPSCs resolve and ~70% of patients may be persistently free (>3 years) of any complication. The most striking demonstration of this difference is made observing a patient who has a penetrating complication at presentation, and then no subsequent complication. The cumulative Montreal phenotype will assign this patient a B3 phenotype for the duration of their disease course. The rolling phenotype initially assigns a B3 phenotype, and then reassigns this patient to a B1 (inflammatory) phenotype after the passage of time without a further complication.

## Conclusion

A rolling phenotype provides insight into the long term burden of IPSCs in patients with Crohn's disease. In this IBD referral centre cohort, at any one time point, approximately 70% of patients had durable (>3 years) freedom from an internal penetrating or stricturing complication. This was true regardless of Montreal behaviour classification within one year of diagnosis. This viewpoint provides patients with Crohn's disease, and the clinicians who manage them, a useful estimation of what complications may be expected from the disease in the long term.

## Supporting information

S1 File Demographic data. (CSV)

S2 File Dataframe of progression of rolling phenotype. (CSV)

S3 File Dataframe of all fistula events. (CSV)

S4 File Dataframe of all stenosis events. (CSV)

S5 File Dataframe of all perforation events. (CSV)

S6 File Dataframe of all performed surgeries. (CSV)
